# Same job, same working conditions? A cross-sectional study to examine the similarities and differences of the working situation in ambulatory and residential youth welfare workers

**DOI:** 10.1186/s12995-024-00419-4

**Published:** 2024-05-17

**Authors:** Maren Kersten, Sylvie Vincent-Höper, Tanja Wirth, Sabine Gregersen, Albert Nienhaus

**Affiliations:** 1 Institution for Statutory Accident Insurance and Prevention in the Healthcare and Welfare Services (BGW), Pappelallee 33/35/37, 22089 Hamburg, Germany; 2 Department of Work and Organizational Psychology, Medical School Hamburg, Hamburg, Germany; 3 Institute for Occupational and Maritime Medicine (ZfAM), University Medical Center Hamburg-Eppendorf (UKE), Hamburg, Germany; 4 Competence Center for Epidemiology and Health Services Research for Healthcare Professionals (CVcare), Institute for Health Services Research in Dermatology and Nursing (IVDP), University Medical Center Hamburg-Eppendorf (UKE), Hamburg, Germany

**Keywords:** Stress, Resources, Working condition, Ambulatory and residential social workers, Health

## Abstract

**Background:**

Employees in social work exhibit high rates of sick leave due to mental health issues. Additionally, work-related demands in youth welfare have increased in recent years. Particularly in light of the escalating shortage of skilled professionals in this field, this trend becomes especially critical. The aim of this study is to systematically examine health-relevant working conditions, coping strategies, and health indicators in youth welfare. A special focus is placed on a differentiated analysis of job-related characteristics in the context of outpatient and residential youth welfare.

**Methods:**

Mean values, standard deviations and the reliability of scales are measured. In addition to descriptive statistics, t-tests for analyzing mean differences, as well as correlation analyses and odds ratios as measures of association, are computed.

**Results:**

A total of *N* = 1044 employees in youth welfare participated in the online survey. Among them, 671 individuals belonged to the field of residential youth welfare, and 373 to outpatient youth welfare. The results indicate that, in youth welfare in general, a variety of emotional, social, qualitative, and quantitative demands exhibit high levels. The comparison between outpatient and residential youth welfare reveals differences in half of the demands. The significant differences are observed for social demands and aggression from clients, which are statistically significant higher in the residential setting. Regarding resources, the most significant difference is observed for autonomy, which is higher in the outpatient setting. Overall, the association patterns reveals more similarities than differences between outpatient and residential settings. In both settings working conditions seem to have deteriorated during the pandemic.

**Conclusions:**

In conclusion, the identified job-related characteristics in outpatient and residential youth welfare exhibit more similarities than differences. Nevertheless, the identified differences provide insights into the specific features of each work context, offering valuable starting points for targeted health promotion in practice.

**Trial registration:**

This trial is recorded at the Hamburg University Ethics Committee (AZ 2022_027).

## Background

Social work is a heterogeneous field of work that encompasses a broad spectrum of possible areas of practice, such as homelessness, disability and family support [1–3]. The role of social work as a profession involves empowering and motivating people to deal with life’s challenges, improving their well-being and assisting them in integrating into societal structures [ [4].

One specific domain within social work is youth welfare services, regarded as essential during the coronavirus pandemic [5].

### Ambulatory and residential youth welfare services

Child and youth welfare are services and tasks for young people and their families. It promotes the development of children and young people into responsible and socially competent individuals. A broad distinction is made between two areas in services for these children: ambulatory and residential youth welfare services. These types of youth welfare services adopt two distinct approaches, which are legally embedded in the German Code of Social Law VIII [6].

Ambulatory youth welfare services provide support and assistance to children and young people in their usual social environment. They generally remain with their families. Assistance is rendered by mobile social professionals in youth welfare services, who come to the families’ homes or operate in other suitable environments.

Residential youth welfare services involve removal of the child or young person from their family and their usual environment. Care is provided in facilities such as children’s and young people’s residential groups and homes.

This measure is taken when problems in the domestic environment can no longer be overcome or if the child is at great risk [7, 8].

The professional background of employees in ambulatory and residential youth welfare services is diverse with regard to professional training, academic studies and additional qualifications. Youth welfare services include social workers, social pedagogues, psychologists, educators, therapists and social scientists. This target group is hereafter referred to as employees in youth welfare services.

### Work situation in youth welfare services

Both forms, ambulatory and residential, share the following working conditions: the job involves direct personal contact, encompassing the task of building relationships with clients [9–11]. Working in youth welfare services entails a high level of emotional strain as employees are constantly faced with their clients’ psychological and physical suffering [12, 13]. Clients have often had traumatic experiences, which can be very emotionally draining for employees in youth welfare services and can lead to emotional exhaustion [14, 15]. Furthermore, there is the worry about potential physically and psychologically violent incidents in the workplace [14, 16, 17].

The work situations differ in the following points: For the inpatient context in particular, it became clear that employees are in contact with young people over a long period of time, who often have a high potential for conflict. Adolescents in the inpatient context often had negative relational and traumatic experiences [18, 19]. Therefore, employees in the context of residential youth welfare services are particularly susceptible to experiencing verbal and physical assaults [20–25]. In addition to client-specific stressors, the job involves shift work, irregular working hours, and demands flexible work schedules from the staff [16, 19, 24, 26].

The work in ambulatory youth welfare services is characterized by high case numbers and a high level of organizational effort [10, 27]. Additionally, employees in outpatient care report a high level of personal responsibility, self-determination, autonomy in their work [28].

### Coping strategies in youth welfare services

Employees in youth welfare services also develop coping strategies to deal with particularly stressful or challenging situations. These strategies may be the patterns of thought or behaviour which people use to find a way out of difficult occupational situations and manage stress [29, 30]. A qualitative and quantitative study of ambulatory youth welfare services showed that employees particularly cited health-endangering coping strategies such as extension of working hours and presenteeism as a response to a demand such as time pressure [31].

### Health situation in youth welfare services

If we look at the health of employees in youth welfare services, adverse psychological stress levels may result in employees developing psychological conditions, such as depression and anxiety disorders, and physical diseases, such as heart and circulatory diseases [15, 32]. What is particularly striking overall is that social occupational groups in health reports have a very high number of days when they are unable to work due to psychological conditions [33, 34]. If we look at the sick certificates for Covid-19, it is evident that educationals and healthcare occupations are the most affected [35]. To gain insights into the subjective experience of social work employees during the coronavirus pandemic, studies were undertaken which examined these aspects [36–38]. The studies confirm that employees in healthcare and welfare, especially women, were more frequently affected by sickness related to Covid-19 than employees in other sectors [34, 39].

### Theoretical framework

As a theoretical framework in this study we used the Job Demands-Resources (JD-R) model [40] to understand the relationship between job characteristics and employee well-being. According to the model, job demands refer to aspects of the job that require sustained physical and/or psychological effort and are associated with physical and psychological costs (e.g. emotional demands, time pressure). On the other hand, job resources are aspects of the job that help employees achieve work goals, reduce job demands, and stimulate personal growth and development (e.g. autonomy, social support). The JD-R model posits that high job demands can lead to strain and burnout, while high job resources can lead to motivation, engagement, and work-related well-being.

### Research questions

In summary, it can be said that educational professionals in the youth welfare sector are confronted with stressful working conditions and a high level of strain. It is evident that mental health needs to be promoted within this occupational group. Having said that, there is comparatively little knowledge about the different working conditions that need to be addressed to improve the mental health of employees in youth welfare services. There are a few studies that focused on selected issues and do not provide an overview of health-relevant working conditions [3, 10, 13].

This study therefore aims to record health-relevant working conditions in youth welfare services and establish links to employees’ indicators of mental health. Besides looking at youth welfare services in general, the study also centres on a differentiated analysis of both ambulatory and residential youth welfare services, because the mentioned findings reveal that the experience of work, e.g. regarding aggression or autonomy, differs within each setting [21, 24, 28, 41].

We believe that the analysis of the work and health situation of employees in youth welfare services on a general and area specific-level will provide information regarding general and specific needs of the employees. We therefore analyse health-relevant working conditions, coping behaviours and mental health indicators of employees in youth welfare services both as a total sample and at a sub-sample level.

The following research questions can be derived from the objective of the study:

1. How do working conditions, coping behavior and health indicators manifest themselves in youth welfare services in general?
2. What working conditions, coping behaviours and health indicators exhibit similar scores in ambulatory and in residential youth welfare services? Where are differences?
3. Are there any differences in working conditions recorded for the current period compared to those before the coronavirus pandemic?
4. How do the relationships between working conditions and health indicators manifest themselves in youth welfare services in general?
5. What relationships are similar in both ambulatory and residential services? Where do differences arise?

By answering these research questions, indications are given as to which specific working conditions or coping behavior can be addressed for workplace health promotion in youth welfare in general and specifically for outpatient and inpatient youth welfare.

## Methods

### Study design and participants

Participants in the study were contacted through associations insured by the Institution for Statutory Accident Insurance and Prevention in the Healthcare and Welfare Services (BGW), through the BGW website and via a BGW online-newsletter. Recruitment for this online-survey took place over 15 days in January 2023.

Participants were asked to give their consent to voluntary participation in the study. The Ethics Committees at the University of Hamburg approved this study (registration number: AZ 2022_027).

### Measuring instrument

A sector-specific questionnaire was adapted to assess the work and health situation in youth welfare services for the cross-sectional study. The questionnaire used in this study was originally developed using a mixed-methods approach in a former pilot study in youth welfare services conducted by Vincent-Hoeper et al. [31] and comprises demands, resources, coping behaviours and health indicators. We expanded the questionnaire in the present study including constructs which record the specific demands during the coronavirus pandemic [42]. To examine the relevance of coronavirus and its possible impact on working conditions more precisely, the expanded survey included questions related to the *emotional demands* and *time pressure*, asking participants to rate their *current* situation and the situation *before* the coronavirus pandemic. Only these two working conditions were chosen for comparison before and after the coronavirus pandemic since research contain information on the possible impact of the coronavirus pandemic on these demands [15, 37, 42]. All other scales were assessed at present only. We also developed two new scales which included questions on fear of coronavirus infection and on work safety measures to protect against coronavirus infections. These aspects were incorporated to assess the impact of the coronavirus pandemic on work in youth welfare services. All scales were listed in Table 1.

**Table 1:** Evaluation scales used in the study

Scale	Number of items	Sample item	Reference
**A. Demands**			
Emotional demands	3	How often is your work highly emotionally demanding? ■ At present ■ * Before the coronavirus pandemic	COPSOQ [43], slightly adapted
Hiding emotions	3	How often does your work require that you hide your feelings?	COPSOQ [43], ISAK-K [44] slightly adapted
Quantitative overload	3	How often are you under time pressure? ■ At present ■ * Before the coronavirus pandemic	COPSOQ [43], slightly adapted
Uncertainty in decision making	3	How often do you have to make decisions without sufficient information?	ISAK-K [44], (1 Item developed by the authors)
Qualitative overload	4	How often do you have to make decisions without sufficient information?	SALSA [45], (1 Item developed by the authors)
Social demands by clients	4	How often do clients have too high expectations on you?	ISAK-K [44], (2 Items developed by the authors)
Aggression by clients	2	Did you experience physical aggression by clients the last 12 month?	Schablon et al. [24]
Sexual harassment	1	How great is the risk of you experiencing sexualized violence or harassment from colleagues or superiors?	developed by the authors
Role Conflict	4	Are contradictory demands placed on you at work?	COPSOQ [43], (2 Items developed by the authors)
Physical work environment	5	Are you affected at work by the following things? ■ noise	SALSA [45]
* Fear of coronavirus infection	5	I am afraid of becoming infected with the coronavirus at my workplace.	developed by the authors
**B. Resources**			
Autonomy	3	The job allows me to plan how I do my work.	WDQ Autonomy [46]
Participation	3	If someone has a good idea, it is possible to put it into practice in this company.	SALSA [45], (1 Item developed by the authors)
Predictability	2	At your place of work, are you informed well in advance concerning for example important decisions, changes, or plans for the future?	COPSOQ [43]
Appreciation	3	Personal engagement and willingness to perform pays off in this organization.	DiGa [47]
Meaning of work	2	Is your work meaningful?	COPSOQ [43]
Feedback/ recognition by the supervisor	3	My supervisor lets me know how well I do my work.	COPSOQ [43]
Fairness/ integrity by the supervisor	3	My supervisor makes sure that the work is fairly distributed among the employees.	GEFA [48]
Social support by the supervisor	3	How much can you rely on your supervisor if problems occur at work?	SALSA [45]
Social support by colleagues	3	How much can you rely on your colleagues if problems occur at work?	SALSA [45]
Social exchange	2	I have the opportunity to meet with other colleagues in my work.	WDQ [46], slightly adapted
*Information on coronavirus at the workplace	3	I am well informed by my institution about the planned and implemented operational measures regarding the coronavirus crisis.	developed by the authors
*Health climate	4	Great importance is attached to employees’ health and well-being in our organization.	Psychosocial Safety Climate [49], slightly adapted
**C. Coping**			
Extension of working hours	4	How often did you make yourself available for your supervisor, colleagues, or clients during leisure time in the last three months?	Krause et al. [50]
Presenteeism	3	How often did you work despite being sick in the last three months?	Krause et al. [50]
**D. Indicators of mental health**			
Job satisfaction	1	Regarding your work in general: How pleased are you with your job as a whole, all things considered?	COPSOQ [43]
Well-being	5	In the last two weeks, I have felt cheerful and in good spirits.	WHO [51]
Depressive symptoms	8	I have sad moods.	Mohr and Müller [52]
Personal Burnout	6	How often do you feel tired?	CBI [53]

*Additions to the pilot study

Socio-demographic data are collected using 12 items. Likert response scales have a range of values from 1 to 5, except for aggression by clients and the well-being scale (ranging from 1 to 6) and the depressive symptoms scale (ranging from 1 to 7) with 1 representing the lowest level in each case.

### Statistical analyses

The mean values, standard deviations and Cronbach’s alpha, which measures reliability, are calculated for the scales to describe the work and health situation in youth welfare services. For scales with less than three items, we provide Pearson correlation instead of Cronbach’s alpha as a measure of reliability [54].

The mean value of all scales and their standard deviations for each group are calculated to provide the comparison of working conditions, coping behaviours and health indicators between ambulatory and residential youth welfare services. The mean values of the scales are compared using t-tests for independent samples. The effect sizes of the mean differences are quantified using Cohen’s d, with values of 0.2 indicating a small effect, values of 0.5 representing a medium effect, and those exceeding 0.8 signifying a strong effect [55].

In order to compare the mean values of the work characteristics at the present time and before the coronavirus pandemic, t-tests were applied to related samples in the respective sub-samples from ambulatory and residential youth welfare services.

To determine relationships between employees’ job characteristics and their health indicators, correlation analyses were calculated using the Pearson correlation coefficient at the overall sample level, as well as and odds ratios at subgroup level based on cross-tabulations using median splits.

## Results

### Descriptive statistics

Table 2 shows the collected sociodemographic variables based on the total sample and the sub-samples. *N* = 1044 youth welfare services employees took part in the cross-sectional survey overall. Of these, 671 individuals (64.3%) belonged to residential youth welfare services and 373 (35.7%) to ambulatory youth welfare services. The average age of participants is 37.6 years of age; in ambulatory youth welfare services the average is 43.2 years of age and 39.2 years in residential services. Within the sample, female participants comprised 282 individuals (75.6%) in ambulatory services and 457 individuals (68.1%) in residential services.

**Table 2:** Descriptive statistics for study variables of Ambulatory and Residential Youth Welfare Workers

Sociodemographic variables		Total *n* (%)	Ambulatory *n* (%)	Residential *n* (%)	Comparison ambulatory vs. residential
Gender	Female	739 (70.8 %)	282 (75.6 %)	457 (68.1 %)	*p* = 0.016
	Male	302 (28.9 %)	89 (23.9 %)	213 (31.4 %)	
	Other	3 (0.3 %)	2 (0.5 %)	1 (0.2 %)	
	Total	1044 (100 %)	373 (35.7 %)	671 (64.3 %)	
Age in years		M 40.63 SD 11.71	M 43.21 SD 11.30	M 39.19 SD 11.69	*p* < 0.001 Cohen’s d = 0.35
Qualification (multiple answers possible)	Social worker	414 (39.7%)	98 (26.3 %)	316 (47.1 %)	*p* < 0.001
	Educator	624 (59.8 %)	282 (75.6 %)	342 (51.0 %)	*p* < 0.001
	Psychologist/ therapist	978 (93.7 %)	330 (88.5 %)	648 (96.6 %)	*p* < 0.001
	Social scientist	1026 (98.3 %)	365 (97.9 %)	661 (98.5 %)	*p* = 0.436
	Other*	890 (85.2 %)	314 (84.2 %)	576 (85.8 %)	*p* = 0.437
Number of years exercising profession		M 13.56 SD 10.24	M 14.92 SD 10.29	M 12.80 SD 10.15	*p* < 0.001 Cohen’s d = 0.21
Contractual working hours (in weekly hours)		M 34.11 SD 6.79	M 32.38 SD 7.38	M 35.07 SD 6.24	*p* < 0.001 Cohen’s d=-0.40
Actual working hours		M 37.24 SD 8.82	M 34.95 SD 9.03	M 38.51 SD 8.45	*p* < 0.001 Cohen’s d=-0.41
Payment for overtime	Yes	485 (46.5%)	149 (40.0 %)	336 (50.0 %)	*p* = 0.007
	No	484 (46.4 %)	194 (52.0 %)	290 (43.0 %)	
	No overtime	75 (7.2 %)	30 (8.0 %)	45 (7.0 %)	
Compensatory time-off for overtime	Yes	902 (86.4 %)	336 (90.1 %)	566 (84.4 %)	*p* = 0.001
	No	102(9.8%)	20 (5.4 %)	82 (12.2 %)	
	No overtime	40 (3.8 %)	17 (4.5 %)	23 (3.4 %)	
Fixed-term contract	Yes	68 (6.5 %)	20 (5.4 %)	48 (7.2 %)	*p* = 0.261
	No	976 (93.5 %)	353 (94.6 %)	623 (92.8 %)	
Management position	Yes	338 (32.4 %)	88 (23.6 %)	250 (37.3 %)	*p* < 0.001
	No	706 (67.6 %)	285 (76.4 %)	421 (62.7 %)	
Supervisor	Yes	1024 (98.1 %)	364 (97.6 %)	660 (98.4 %)	*p* = 0.382
	No	20 (1.9 %)	9 (2.4)	11 (1.6 %)	

*Including systemic therapist/counsellor, remedial teacher, trauma educator, educationalist, remedial therapist

The average length of employment is 14.9 years in ambulatory services and 12.8 years in residential services. The vast majority of participants, 353 individuals (94.6%) in ambulatory services and 623 individuals (92.8%) in residential services, have a permanent employment contract. Employees in ambulatory youth welfare services are contracted to work an average of 32.4 h a week and those in residential services 35.1 h per week. The reported actual working hours per week is 35 h in ambulatory services and 38.5 h in residential services. 88 individuals (23.6%) in ambulatory services and 250 individuals (37.3%) in residential services held a management position. 97.6% of the respondents in ambulatory services were under the supervision of a manager while it was 98.4% in residential services.

A comparison of the employees’ sociodemographic variables in both ambulatory and residential youth welfare services showed that the two sub-samples differed significantly from one another with regard to the majority of characteristics. There are no significant differences solely with regard to social scientists’ qualifications, other qualifications, temporary employment, and whether employees have a supervisor or not.

### Mean value comparisons, t-tests and effect sizes

The descriptive statistic for demands, resources, coping behaviours and indicators of mental health in youth welfare services is presented in Table 3.

**Table 3:** Demands, resources, stress management strategies and mental health indicators of employees in youth welfare services

	*N*	M	SD	α	range
A. Demands					
Emotional demands	1044	3.23	0.69	0.78	1–5
Emotional demands (before coronavirus)	1027	3.08	0.73	0.78	1–5
Hiding emotions	1044	2.94	0.82	0.75	1–5
Time pressure	1044	3.33	0.85	0.84	1–5
Time pressure (before coronavirus)	1024	3.24	0.85	0.84	1–5
Uncertainty	1044	2.83	0.72	0.68	1–5
Qualitative demands	1044	3.02	0.72	0.67	1–5
Social demands by clients	1044	3.37	0.71	0.80	1–5
Aggression by clients	1044	2.78	1.34	0.70^1^	
Role conflicts	1044	2.77	0.73	0.83	1–5
Physical work Environment	1044	2.50	0.77	0.72	1–5
Fear of coronavirus	1044	2.34	1.08	0.84	1–5
B. Resources					
Autonomy	1044	3.73	0.80	0.74	1–5
Participation	1044	3.11	0.80	0.78	1–5
Predictability	1044	3.67	0.93	0.61^1^	1–5
Organisational appreciation	1044	2.88	0.78	0.69	1–5
Meaningful work	1044	4.34	0.68	0.72^1^	1–5
Health-promoting leadership behaviour	1024	3.38	0.96	0.91	1–5
Social support by the supervisor	1024	3.83	0.95	0.91	1–5
Social support by colleagues	1044	3.95	0.82	0.88	1–5
Social exchange in teams	1044	3.82	0.90	0.69^1^	1–5
Coronavirus workplace safety	1044	3.75	0.86	0.77	1–5
Organisational health climate	1044	3.35	0.86	0.87	1–5
C. Coping					
Extension of working hours	1044	3.35	1.03	0.82	1–5
Presenteeism	1044	2.75	1.20	0.85^1^	1–5
D. Indicators of mental health					
Job satisfaction	1044	3.70	0.82	-	1–5
Well-being	1044	3.41	1.01	0.86	1–6
Depressive symptoms	1044	2.69	1.01	0.87	1–7
Personal burnout	1044	2.92	0.82	0.90	1–5

^1^item-total-correlation (r_it_)

The Cronbach’s alpha ranged between α = 0.69 and α = 0.91 and largely exhibit good or acceptable characteristic values. The intercorrelation between the items on the scales with two items is r_it_ =0 0.70 (aggression), r_it_ = 0.61 (predictability), r_it_ = 0.72 (meaningful work), r_it_ = 0.69 (social exchange in teams) and r_it_ = 0.85 (presenteeism), which is satisfactory. Job satisfaction is a single-item measure for which reliability cannot be calculated.

Employees’ responses in the total sample are illustrated using percentage values to provide a better classification of scale scores. To highlight working conditions with particularly high manifestations, we defined an agreement rate. The percentages in the two highest response categories 4 (often) and 5 ([almost] always) were combined for this purpose. The percentage values are presented for the scales reporting agreement of ≥ 25% combined in these two highest response categories.

The following six demands exhibit a high or very high score: Emotional demands at present (37%) and before the coronavirus pandemic (30%), qualitative demands (32%), social demands (54%), and time pressure at present (43%) and before the coronavirus pandemic (39%).

Overall, the resources are rated quite highly, with the top four being: meaningful work (92%), social exchange in teams (74%), social support from colleagues (73%) and social support from the supervisor (68%).

When it comes to coping strategies, it is evident that 44% of the surveyed employees rated the characteristic of extension of working hours and 33% rated presenteeism with *often* and *(almost) always*.

When it comes to health indicators, only personal burnout was perceived by 27%, more than a quarter, in the two highest categories (often and [almost] always).

In the next step, we analysed similarities and differences regarding residential und ambulatory youth welfare services. Table 4 displays the scales with significant mean differences. All other scales show no significant differences.

**Table 4:** Differences in mean values in ambulatory and residential youth welfare services

	Ambulatory youth welfare services			Residential youth welfare services			*p*-value	Cohen’s d
	*N*	M	SD	*N*	M	SD		
Time pressure	373	3.21	0.90	654	3.39	0.82	0.002	-0.21
Uncertainty	373	2.76	0.71	671	2.87	0.72	0.014	-0.16
Qualitative demands	373	2.92	0.69	671	3.07	0.73	0.001	-0.21
Social demands by clients	373	3.13	0.68	671	3.51	0.69	< 0.001	-0.56
Aggression by the clients	373	2.25	1.22	671	3.07	1.32	< 0.001	-0.64
Autonomy	373	4.00	0.67	671	3.57	0.78	< 0.001	0.58
Meaningful work	373	4.25	0.69	671	4.38	0.67	0.004	-0.19
Organisational health climate	373	3.50	0.90	671	3.27	0.86	< 0.001	0.27
Extension of working hours	373	2.69	1.00	671	3.53	0.91	< 0.001	-0.90
Presenteeism	373	2.35	1.09	671	2.98	1.20	< 0.001	-0.54

Overall, the mean comparisons show that five of the ten recorded demands exhibit significant differences in their mean value. In the case of resources, three of the eleven recorded resources exhibit significant differences. In the case of coping behaviours, both behaviour patterns differ significantly. The health indicators do not show any significant differences. Table 4 contains the values for the scales where differences have arisen.

While five of the ten demands present similar scores, the following demands in residential youth welfare services have significantly higher scores than in ambulatory services: *Time pressure* (*p* = 0.002; *d* = -0.21), *uncertainty* (*p* = 0.014; *d* = -0.16), *qualitative demands*, (*p* = 0.001; *d* = -0.21), *social demands* (*p* < 0.001; *d* = -0.56) and *aggression by the clients* (*p* < 0.001; *d* = -0.64).

Of the eleven recorded resources, eight scores show similar results in both ambulatory and residential services. In contrast, the resources *autonomy* (*p* < 0.001; *d* = 0.58) and *organisational health climate* (*p* < 0.001; *d* = 0.27) are statistically significantly greater in ambulatory youth welfare services. The resource *meaningful work* (*p* = 0.004; d = -0.19) scores somewhat higher in residential youth welfare services.

The biggest difference in resources is observed in autonomy. This is significantly greater in ambulatory youth welfare services with a medium effect size.

There are clear differences in health-endangering stress management behaviours on close examination. Both *extension of working hours* (*p* < 0.001; d = -0.90) and *presenteeism* (*p* = 0.001; d = -0.54) score significantly higher in residential youth welfare services. The effects may be categorised as medium to high. No statistically significant differences were evident in the four mental health indicators (job satisfaction, well-being, depressive symptoms, personal burnout).

The rating for the job characteristics *emotional demands* and *time pressure* before and after the coronavirus are listed in Table 5.

**Table 5:** Comparison of job characteristics before and after coronavirus in ambulatory and residential youth welfare services

	Ambulatory youth welfare services				Residential youth welfare services			
	*N*	M	SD	α	*N*	M	SD	α
Emotional demands - at present	373	3.20	0.67	0.78	671	3.24	0.71	0.78
- before the coronavirus Pandemic	371	3.06	0.68	0.77	656	3.09	0.75	0.78
*p*-value		< 0.001				< 0.001		
Cohen’s d		0.26				0.27		
Time pressure - at present	373	3.22	0.90	0.85	654	3.40	0.82	0.84
- before the coronavirus Pandemic	370	3.13	0.86	0.84	671	3.30	0.84	0.84
*p*-value		0.002				< 0.001		
Cohen’s d		0.16				0.20		

Employees in both ambulatory and residential youth welfare services perceived *emotional demands* and *time pressure* to be greater after the coronavirus pandemic than before the pandemic. The effects for the differences in mean values fall within the lower range.

### The relationships between work characteristics employees’ health

All correlations of working conditions and health indicators are significant (Table 6). The analyses show that the demands correlate negatively with the positive health indicators (job satisfaction and well-being) and positively with the negative health indicators (personal burnout and depressive symptoms). As expected, the resources correlate positively with the positive indicators and negatively with the negative indicators of mental health. Almost all scales show correlations of *r* ≥ 0.25 with at least one of the health indicators. Only the scale *aggression by clients* did not meet the criterion of *r* ≥ 0.25 with one of the health indicators.

**Table 6:** Correlation table of work characteristics regarding employees’ health in youth welfare services

	*N*	Job satisfaction	Well-being	Depressive symptoms	Personal burnout
**A. Demands**					
Emotional demands	1044	-0.36***	-0.39***	0.36***	0.46***
Hiding emotions	1044	-0.34***	-0.30***	0.30***	0.32***
Time pressure	1044	-0.32***	-0.32***	0.19***	0.34***
Uncertainty	1044	-0.37***	-0.29***	0.32***	0.32***
Qualitative demands	1044	-0.36***	-0.34***	0.33***	0.40***
Social demands by clients	1044	-0.29***	-0.23***	0.18***	0.27***
Aggression by clients	1044	-0.23***	-0.13***	0.12***	0.17***
Role conflicts	1044	-0.43***	-0.33***	0.33***	0.36***
Physical work Environment	1044	-0.31***	-0.27***	0.24***	0.30***
Fear of coronavirus	1044	-0.16***	-0.18***	0.22***	0.25***
**B. Resources**					
Autonomy	1044	0.39***	0.29***	-0.25***	-0.27***
Participation	1044	0.51***	0.32***	-0.26***	-0.26***
Predictability	1044	0.49***	0.31***	-0.27***	-0.28***
Organisational appreciation	1044	0.28***	0.22***	-0.26***	-0.19***
Meaningful work	1044	0.52***	0.40***	-0.30***	-0.34***
Health-promoting leadership behaviour	1024	0.47***	0.28***	-0.24***	-0.24***
Social support by the supervisor	1024	0.46***	0.28***	-0.24***	-0.23***
Social support by colleagues	1044	0.38***	0.27***	-0.26***	-0.22***
Social exchange in teams	1044	0.40***	0.26***	-0.25***	-0.27***
Coronavirus workplace safety	1044	0.33***	0.18***	-0.21***	-0.18***
Organisational health climate	1044	0.54***	0.37***	-0.25***	-0.31***
**C. Coping**					
Extension of working hours	1044	-0.26***	-0.24***	0.16***	0.29***
Presenteeism	1044	-0.33***	-0.35***	0.26***	0.44***

Remarks *N* = 1044. * *p* < 0.05 ** *p* < 0.01 *** *p* < 0.001

It should be noted that in field studies exploring links between job characteristics and workers’ indicators of physical and mental health, the maximum correlations typically range between about *r* = 0.20 and *r* = 0.30 [56]. Therefore, correlations of *r* = 0.25 or more are viewed as relevant because they make a significant contribution towards explaining the state of health associated with work.

To examine the relationships between the work characteristics and the mental health indicators more closely, we conducted multiple hierarchical regression analyses. Due to the high number of predictors and potential issues with multicollinearity when interpreting the regression coefficients, it is important to recognize that the work characteristics are not entirely independent from each other. Instead, they may share variance components in the dependent variable. This can lead to suppressor effects, hence we refrain from interpreting and reporting the regression weights. Nevertheless, since multicollinearity does not affect variance explanation, we provide the explained variance. Initially, we controlled for ambulatory versus residential youth welfare services, gender, age, working hours per week, and fixed-term versus permanent contract. Regarding personal burnout, the control variables, with only age showing significance, explain 3.8% of the variance. Demands accounted for an additional 28.6%, resources for 3.3%, and coping strategies for 5%.

For depressive symptoms, the control variables, with age and gender showing significance, explain 2% of the variance. Demands accounted for an additional 22.3%, resources for 5.3%, and coping strategies for 1.8%.

With regard to the outcome *personal burnout*, demands in ambulatory youth welfare services exhibit ORs between 1.12 and 2.95 while the OR in residential services ranges between 1.70 and 4.13. The OR are similarly high in both services for most characteristics, except for the scales emotional demands, qualitative demands as well as social demands by clients, and role conflict, where residential services exhibit substantially higher values. In ambulatory services, the ORs for resources are between 0.41 and 0.78 while they range from 0.35 to 0.63 in residential services. Overall, the ORs in the two areas do not differ significantly from each other. The results are presented in Table 7 (personal burnout) and Table 8 (Depressive symptoms).

**Table 7:** Odds ratio for the relationship between job characteristics and personal burnout

	Ambulatory youth welfare services		Residential youth welfare services		
	OR	95% CI	OR	95% CI	
Emotional demands	2.78	(1.83–4.24)	4.13	(2.98–5.71)	
Hiding emotions	2.44	(1.59–3.74)	2.67	(1.95–3.66)	
Time pressure	2.89	(1.89–4.39)	2.68	(1.95–3.68)	
Uncertainty	2.82	(1.83–4.32)	2.82	(2.06–3.87)	
Qualitative demands	2.95	(1.93–4.59)	3.75	(2.70–5.19)	
Social demands by clients	1.87	(1.22–2.86)	2.46	(1.79–3.38)	
Aggression by clients	1.12	(0.74–1.71)	1.70	(1.23–2.37)	
Role conflicts	2.53	(1.66–3.85)	3.20	(2.32–4.41)	
Physical work environment	2.05	(1.35–3.10)	2.05	(1.50–2.82)	
Autonomy	0.57	(0.37–0.89)	0.60	(0.44–0.82)	
Participation	0.51	(0.32–0.79)	0.49	(0.35–0.69)	
Predictability	0.41	(0.27–0.62)	0.37	(0.27–0.51)	
Meaningful work	0.78	(0.52–1.18)	0.63	(0.46–0.86)	
Organisational appreciation	0.42	(0.28–0.64)	0.35	(0.25–0.48)	
Health-promoting leadership behaviour	0.68	(0.45–1.02)	0.57	(0.42–0.77)	
Social support from supervisor	0.51	(0.34–0.77)	0.48	(0.35–0.66)	
Social support from colleagues	0.47	(0.31–0.73)	0.56	(0.40–0.76)	
Social exchange in teams	0.49	(0.32–0.75)	0.41	(0.30–0.57)	
Coronavirus work safety	0.53	(0.35–0.80)	0.60	(0.44–0.81)	
Organisational health climate	0.43	(0.28–0.66)	0.36	(0.26–0.49)	
Extension of working hours	2.32	(1.48–3.66)	2.54	(1.82–3.54)	
Presenteeism	4.91	(3.07–7.86)	2.05	(1.72–2.43)	

**Table 8:** Odds ratio for the relationship between job characteristics and depressive symptoms

	Ambulatory youth welfare services		Residential youth welfare services	
	OR	95% CI	OR	95% CI
Emotional demands	1.80	(1.19–2.72)	2.51	(1.84–3.44)
Hiding emotions	2.10	(1.37–3.21)	2.64	(1.73–3.23)
Time pressure	1.56	(1.04–2.36)	1.30	(0.95–1.77)
Uncertainty	3.14	(2.03–4.90)	2.68	(1.96–3.66)
Qualitative demands	3.17	(2.07–4.90)	2.74	(1.99–3.78)
Social demands by clients	1.43	(0.94–2.19)	1.65	(1.21–2.26)
Aggression by clients	1.65	(1.08–2.53)	1.36	(0.98–1.89)
Role conflicts	2.04	(1.35–3.10)	2.99	(2.17–4.10)
Physical work environment	1.88	(1.24–2.85)	1.98	(1.45–2.71)
Autonomy	0.61	(0.40–0.95)	0.51	(0.37–0.69)
Participation	0.49	(0.31–0.76)	0.52	(0.38–0.72)
Predictability	0.47	(0.31–0.72)	0.43	(0.31–0.58)
Meaningful work	0.54	(0.36–0.82)	0.52	(0.38–0.71)
Organisational appreciation	0.30	(0.20–0.47)	0.53	(3.89–0.72)
Health-promoting leadership behaviour	0.57	(0.38–0.86)	0.47	(0.34–0.63)
Social support from supervisor	0.49	(0.32–0.74)	0.65	(0.54–0.79)
Social support from colleagues	0.34	(0.22–0.54)	0.72	(0.60–0.87)
Social exchange in teams	0.64	(0.42–0.98)	0.43	(0.32–0.60)
Coronavirus work safety	0.42	(0.28–0.64)	0.58	(0.43–0.80)
Organisational health climate	0.43	(0.28–0.66)	0.53	(0.39–0.72)
Extension of working hours	1.68	(1.08–2.63)	1.29	(0.92–1.72)
Presenteeism	2.96	(1.89–4.63)	1.82	(1.33–2.48)

The two coping behaviours extension of working hours and presenteeism exhibit ORs of 2.32 and 4.91, respectively, in ambulatory services and 2.54 and 2.05 in residential services. A higher rating regarding the characteristic presenteeism is observed in ambulatory services compared to residential services.

With regard to the outcome *depressive symptoms* demands in ambulatory youth welfare services exhibit ORs between 1.43 and 3.17 while the OR in residential services ranges between 1.30 and 2.99. For most characteristics, the OR is similarly high in both services, except for the role conflict scale, where residential services exhibit significantly higher values. In ambulatory services, the ORs for resources are between 0.30 and 0.61 while they range from 0.43 to 0.72 in residential services. Overall, the ORs for the resources in the two areas do not differ substantially from each other.

The two coping behaviours extension of working hours and presenteeism exhibit OR values of 1.68 and 2.96 in ambulatory services respectively 1.29 and 1.82 in residential services. Especially, a higher OR value regarding the characteristic presenteeism is observed in ambulatory services compared to residential services.

These odds ratios (OR) illustrate the likelihood of an event occurring compared to the likelihood of it not occurring. The results reveal that in ambulatory youth welfare services, when faced with high qualitative demands, time pressure, uncertainty, and emotional demands, the likelihood of developing personal burnout is nearly three times higher.

For employees in residential youth welfare services, the likelihood of developing personal burnout is over four times higher when emotional demands are high, over three times higher with high qualitative demands or role conflicts, and nearly three times higher with high uncertainty.

The odds ratios between ambulatory and residential settings do not vary significantly, except for emotional demands and presenteeism. In these cases, the results suggest that the likelihood of developing personal burnout is nearly five times higher in the ambulatory setting compared to two times in the residential setting.

Regarding the development of depressive symptoms, employees in ambulatory youth welfare services are over three times more likely to experience them when faced with high qualitative demands and uncertainty. Conversely, in the residential context, the likelihood of developing depressive symptoms is almost three times higher with high role conflicts and qualitative demands. High presenteeism increases the likelihood of developing depressive symptoms by almost three times in the ambulatory setting, whereas in the residential setting, it is less than two times as likely.

## Discussion

The main objective of this study was to systematically investigate health-relevant working conditions, coping strategies and health indicators in both youth welfare services overall and in the subgroups of ambulatory and residential youth welfare services. The knowledge generated in this way about similarities and differences in outpatient and inpatient youth welfare enables the derivation of tailored prevention measures. Thus, one key added value of the study lies in the differentiated analysis and the consequent derivation of specific recommendations for interventions in ambulatory and residential youth welfare services.

### Interpretation of the results

The following conclusions can be drawn with regard to the five questions examined:

Emotional, social, qualitative and quantitative demands show the highest levels in youth welfare. As a consequence, they are considered to be highly relevant. This is consistent with the findings made by Poulsen [10] and Baldschun et al. [13].

The resources achieved high scores overall, particularly meaningful work, social exchange in teams, social support from colleagues and from superior. One third of the employees often adopt self-endangering coping strategies such as extension working hours and presenteeism. This indicates that these dysfunctional coping strategies are frequently exhibited in the area of youth welfare. The two negative health indicators were selected because they have comparatively high values in the youth welfare professional group [10, 15, 57].

The answer to the second question reveals that specific demands exhibit significant differences in scores between ambulatory and residential youth welfare services.

While half of the ten demands show similar scores, quantitative and qualitative demands as well as social demands and aggression by clients are more pronounced in residential youth welfare services. The most significant difference is observed for demands originating from the clients’ behaviour, such as aggression social demands.

Other studies also confirm the heightened perception of aggression in residential services [17, 19]. One explanation for this could be that clients in residential services are individuals with particularly challenging behaviors [8]. Another explanation could be that when young people are in a safe environment (residential youth care), they show their pent-up frustration, fear and violence.

Of the eleven recorded resources, eight show similar results in both ambulatory and residential services. In contrast, the resources autonomy and organisational health climate are statistically significantly greater in ambulatory youth welfare services. The resource meaningful work scores somewhat higher in residential youth welfare services.

The identified effect sizes are low. The biggest difference in resources is observed in autonomy.

There are clear differences in health-endangering coping behaviours. Both extension of working hours and presenteeism score significantly higher in residential youth welfare services. One reason for the higher score may be that employees in residential youth welfare services do not want to let their colleagues on-site down and therefore tend to sacrifice their free time or come to work even when they are unwell. There were no differences in the four health indicators.

The answer to the third question shows that emotional and quantitative demands have increased after the pandemic. This finding indicates that the coronavirus pandemic may have been a catalyst behind the perceived increase. Studies from the coronavirus pandemic period also point in this direction [37, 39, 42]. However, the effects were small what may be due to the fact that these demands had already reached a high level before the pandemic [10, 13, 58].

The answer to the fourth question shows that all correlations between working conditions and employee health are highly significant and indicate substantial correlations in their magnitude. This confirms the health-relevance of these working conditions as stated by Vincent-Hoeper et al. [31].

In line with theoretical assumptions, demands are strongly associated with higher impairments to well-being while resources are primarily linked to a more positive sense of well-being [59].

The response to the fifth question shows that the odds ratios (OR) in both services are generally similar and exhibit only a few deviations. When it comes to personal burnout, the values from the scales for emotional, qualitative and social demands as well as role conflict indicate higher associations with impaired well-being in residential services. With regard to depressive symptoms only role conflict shows a stronger association in residential services than in ambulatory services. Differences with regard to presenteeism are particularly evident in the subgroups. The values for presenteeism suggest that the relation is greater in ambulatory services. What is particularly interesting about this result is that the mean value of presenteeism is higher in inpatient youth welfare than in outpatient youth welfare. However, the negative effects on personal burnout and depressive symptoms are more pronounced in outpatient youth welfare. This may be related to the fact that there is no on-site support from colleagues in the ambulatory context. In outpatient youth services (e.g. home visits, personal counseling sessions), employees tend to be left to their own devices.

### Limitations and implications for research

Our findings may be limited in several ways. First, the cross-sectional design can describe associations, but will never link causation. Second, the explored data is based on a convenience sample that is liable to selection bias. However, it has several positive aspects concerning complete rates [60]. The results of this study are based on a survey of more than 1000 employees. Even though this is a relatively large sample, we are unable to comment on its representativeness because we lack information about the overall population, which may limit the generalisability of the results.

In the present cross-sectional study, working conditions and indicators of mental health were recorded by the same person in the same questionnaire, which can lead to a common method bias.

Especially the comparative results provide some indications of specific approaches to reduce stress on employees in ambulatory and residential youth welfare services. However, a comparison of the sociodemographic variables for both subgroups revealed a number of differences, especially with regard to age, gender, qualifications, working hours, handling overtime and supervisory positions. It cannot be excluded that these differences are also responsible for some of the differences in the work-related variables.

To highlight working conditions with particularly high manifestations, we defined an agreement rate.

The percentages in the two highest response categories were combined for this purpose and the percentage values are presented for the scales reporting agreement of ≥ 25% combined in these two response categories. This cut-off value of ≥ 25% was determined by us, because there were no indications in literature. This can certainly be viewed critically.

The comparison of job characteristics before and after the coronavirus pandemic comprises a subjective, retrospective evaluation which may be distorted given that a number of years have passed since the period preceding the coronavirus pandemic.

In this study, we assessed emotional demands at a global level because the aim was to obtain an overview of various stressors and resources. We cannot make any statements about the specific stressful emotional experiences underlying them. As this stressor has proven to be particularly relevant, future studies should investigate the phenomenon of emotional demands in more depth. Concepts, such as compassion fatigue vicarious trauma, secondary trauma, and critical incident stress might give significant insights [61–66]. A promising approach in overcoming compassion fatigue, effectively managing the risk of vicarious trauma is a focus on self-care, since studies suggest that employees who regularly engage in self-care activities were less affected by burnout and secondary traumatic stress [67–69].

Furthermore, we only assessed two coping styles related to dysfunctional self-endangering coping behaviour. In future studies, it would be interesting to also measure functional health-promoting coping styles to explore potential explanations of how these coping styles may assist employees in managing their demands more effectively.

An interesting finding pertains to gender and age as significant predictors in the multiple regression analysis, particularly in relation to depressive symptoms. From this, we deduce that sensitivity analyses for specific groups would yield additional insights.

### Implications for practice

The findings suggest that several demands and resources are relevant in both residential and ambulatory contexts, such as emotional demands or social support. Furthermore, we identified specific aspects that could be targeted to enhance employee health within each context. In residential settings, higher levels of stressors including time pressure, uncertainty, qualitative demands, social demands, and aggression by clients were observed. Addressing these stressors presents a promising avenue for health promotion in residential settings. Additionally, resources such as autonomy and organizational health climate were found to be less prominent in residential youth welfare services compared to ambulatory settings. Therefore, efforts to promote these resources should be prioritized in workplace health promotion initiatives in the residential setting.

Another notable finding concerns health-endangering stress management behaviors, such as extended working hours and presenteeism, which are more prevalent in residential youth welfare services. Specifically, presenteeism should be addressed more effectively in the residential context due to its detrimental effects on mental health.

## Conclusion

Previous studies revealed a deficit in systematic examination of working conditions in youth welfare services [3]. Thus, the present study aimed to provide a systematic and differentiated examination of health-relevant demands and resources in youth welfare services in general and specifically in the subgroups of ambulatory and residential youth welfare services. On the whole, there are more similarities than differences between the two services. However, there are quite pronounced differences with regard to number of characteristics (e.g., clients’ related demands and presenteeism). These differences provide new information for main focuses in workplace health promotion.

Accordingly, this study delivers a more promising contribution for custom-fit prospective and preventive health promotion in ambulatory and residential youth welfare services.

## Data Availability

Data is available only in German.
